# Oral Health Service Use in Older Peruvians Before and During the COVID-19 Pandemic

**DOI:** 10.1016/j.identj.2023.12.003

**Published:** 2024-01-14

**Authors:** Diego Azañedo, Fabriccio J. Visconti-Lopez, Akram Hernández-Vásquez

**Affiliations:** a Universidad Científica del Sur, Lima, Peru; b Sociedad Científica de Estudiantes de Medicina–UPC, Lima, Peru; c Centro de Excelencia en Investigaciones Económicas y Sociales en Salud, Vicerrectorado de Investigación, Universidad San Ignacio de Loyola, Lima, Peru

**Keywords:** Oral health, Health services accessibility, Health care disparities, Aged, COVID-19, Peru

## Abstract

**Objectives:**

The aim of this work was to analyse inequalities in oral health services utilisation (OHSU) in older Peruvian adults through comparative analysis of the years 2019 and 2021.

**Methods:**

We conducted a secondary analysis of data from the 2019 and 2021 Demographic and Health Survey (ENDES). The outcome variable was OHSU by older Peruvian adults in the past year. We used Poisson generalised linear models adjusted for age and sex to assess changes in OHSU by sociodemographic characteristics. The Erreygers concentration index was used to describe the socioeconomic inequalities in OHSU. The contribution of each variable to inequalities was estimated by a decomposition analysis.

**Results:**

In 2021, OHSU probability amongst older Peruvian adults decreased by 37% compared to 2019. The decline was greatest in those aged 80 or older (51%), the lowest wealth quintile (47%), those with functional limitations (53%), and those whose native language is Quechua or other indigenous languages (47%). Surprisingly, we observed a reduction in OHSU inequalities (difference: −0.1074; *P* = .003) during the COVID-19 pandemic, particularly amongst rural residents (difference: −0.0771; *P* = .030), the lowest wealth quintile (difference: −0.0764; *P* = .020), and those with functional limitations (difference: −0.3665; *P* < .001). Poverty accounted for 73% of the inequality in 2021.

**Conclusions:**

The probability of OHSU has significantly decreased likely due to the COVID-19 pandemic. Paradoxically, we observed a reduction in OHSU inequalities during the pandemic, despite the known socioeconomic impact. However, further research is required to gain deeper understanding of this phenomenon.

## Introduction

The burden of oral diseases is a global public health problem affecting individuals of all ages and socioeconomic levels.^1^^,^^2^ According to the World Health Organization's Global Report on Oral Diseases, in 2017, almost half of the world's population (3.5 billion) experienced some form of oral disease, and 84% of those affected lived in low- and middle-income countries.^1^^,^^3^ Older adults are one of the most affected and vulnerable groups with regard to these diseases, with peaks of periodontal disease prevalence (around 30%) at the age of 60 years and peaks of edentulism prevalence (ranging from 12% to 40%) between 60 and 95 years.^1^^,^^3^ In Peru, the prevalence of edentulism in individuals aged 60 years or older is amongst the highest in the world, estimated to be between 34.1% and 50.9% in 2019.^3^ Deteriorated oral health in older adults can lead to pain, discomfort, and infections, negatively impacting their quality of life as well as their general, nutritional, and social health.^4^, ^5^, ^6^, ^7^ As the proportion of the older adult population increases worldwide, it is essential for health care systems to be adequately equipped to provide timely oral health care to meet their needs and limit complications and more complex and costly treatments in the future.^8^^,^^9^

Access to oral health services is a crucial component of preventing, treating, and rehabilitating oral diseases. However, the COVID-19 pandemic has had a significant impact on oral health services utilisation (OHSU), affecting even the elderly population in developed countries.^10^^,^^11^ In Peru, the prevalence of OHSU in the last 6 months for people older than 60 years decreased from 21.8% in 2019 to 14.4% in 2021.^12^^,^^13^ Although there was a reduction of just over 7 percentage points in the prevalence of OHSU in this population, it is unknown whether there are groups of the population that are more affected, such as the rural population, persons without health insurance, or individuals with functional limitations, given the heterogeneity of the Peruvian population.

Inequalities in OHSU can exacerbate oral health problems in vulnerable and economically disadvantaged population.^14^ COVID-19 has likely exacerbated these disparities by restricting access to oral health services, as seen with children younger than 12 years.^15^ This puts those already at a disadvantage at greater risk for poor oral health. In Peru, public health insurance does not cover many necessary dental treatments for older adults, making it challenging for them to pay out-of-pocket, which negatively impacts household economies.^16^, ^17^, ^18^ Despite the high prevalence of oral health problems in the elderly population and the potential impact of the pandemic, there has been no assessment of OHSU inequalities amongst older adults in Peru.

The objective of this study is to examine changes in OHSU amongst older Peruvian adults using data from the Demographic and Family Health Survey (ENDES) in 2019 and 2021, as an approximation of the impact evaluation of the COVID-19 pandemic. The study results will provide valuable insights to national health policymakers on the need to redesign oral public health strategies that can better serve the vulnerable and socially disadvantaged populations. Additionally, the findings may serve as a foundation for developing more accessible, inclusive, and targeted strategies to improve oral health in older Peruvian adults.

## Materials and methods

### Data source and study design

Our secondary data source was the ENDES for the years 2019 and 2021, collected through a cross-sectional study design. Specifically, these years were selected considering the most recent year available in the ENDES and the year prior to the COVID-19 pandemic. The ENDES is a nationally representative cross-sectional health survey carried out annually in Peru since 1986 by the National Institute of Statistics and Informatics (INEI, abbreviation in Spanish).^19^^,^^20^ The sampling design was probabilistic, 2-stage, and stratified by department and rural-urban area to obtain a nationally representative sample.^19^^,^^20^ ENDES aims to collect demographic, health, and socioeconomic data at the national level, with data obtained through interviews conducted by trained personnel in the participants' households. The survey includes a variety of topics, such as health, education, housing, and employment, amongst others, and are administered using structured questionnaires.^19^^,^^20^

The questionnaires, selection of participants, and survey implementation are standardised to ensure that the results from each year of the ENDES are comparable. A detailed description of participant recruitment, survey design, and data collection procedures is available in the technical report of the ENDES 2019 and 2021.^19^^,^^20^ The datasets obtained for this study are publicly available on the microdata webpage of the INEI: https://proyectos.inei.gob.pe/microdatos/.

This manuscript was written following the guidelines of the Strengthening the Reporting of Observational Studies in Epidemiology (STROBE) statement.

### Variables and measurements

#### Outcome variable

The study's outcome variable was OHSU amongst older Peruvian adults, coded as either 1 (if the older adult used oral health services within the last 12 months) or 0 (if not).

#### Socioeconomic status

The variable used to assess inequality in this study was the wealth quintile based on the wealth index. The wealth index in the ENDES is derived from household characteristics and assets using a principal component analysis as described by Rutstein and Johnson.^21^ In our study, the "wealth index" was categorised into 5 groups or quintiles, which means that the participants are divided into 5 categories according to the index obtained, ranging from the lowest quintile (the 20% with the lowest index) to the highest quintile (the 20% with the highest index).

#### Explanatory variables

The selection of possible explanatory variables was based on the PROGRESS-Plus framework.^22^ The variables available in ENDES were age group (60–69, 70–79, ≥80 years), sex (female, male), education level (up to initial education, primary, secondary, higher), area of residence (urban, rural), region of residence (Metropolitan Lima, rest of the coast, highlands, jungle), wealth quintile (richest, richer, middle, poorer, poorest), limitation (yes, no), language learning in childhood (Spanish/Portuguese/other foreign languages, Quechua or native language), and health insurance (yes, no). In Peru, with the implementation of universal health insurance in 2009, oral health care is covered by public health insurance. Peruvian health insurance is structured as follows^23^: (1) Public Integral Health Insurance (SIS), which offers health care services to low-income individuals; (2) Social Security Health Insurance (EsSalud), providing health care services to employed workers and their dependents; (3) Armed Forces and Police Health System, which brings health care to employees of these institutions and their dependents; and (4) private insurance, for individuals who can afford private insurance. Based on these distinct subsystems, we categorised this variable as yes if having any of them and as no in the absence of insurance.

### Statistical analysis

Data were analysed using Stata version 17 (StataCorp, College Station, TX, USA). Prior to analysis, the "svy" command was used to include the sample design and weights of ENDES. Statistical significance was determined using a 2-tailed *P* value < .05.

We used a descriptive analysis to report the frequency distribution and 95% confidence intervals (CIs) of the variables of interest. To estimate prevalence ratios and 95% CI of OHSU at the overall level and according to sample characteristics in 2021, we applied generalised linear models of the Poisson family, with a logarithmic link function, adjusted for age and sex. We used 2019 as a reference year and followed previously reported methodology.^24^ These methods allowed us to compare the OHSU between the 2 survey years and identify potential changes in access to oral health services amongst older Peruvian adults. To interpret these prevalence ratios, we applied a Bonferroni correction procedure to adjust the *P* value for multiple comparisons, establishing a significance level of .002. This cutoff was determined by considering the 26 distinct categories of variables subjected to hypothesis testing to evaluate changes in OHSU. The calculation of the alpha correction adhered to the following formula:

$$Bonferroni^{\prime }s\;corrected\;\alpha =\frac{prespecified\;\alpha \;value}{number\;of\;tests}\;=\;\frac{0.05}{26}\;=0.002$$

Concerning inequality analysis, Erreygers concentration indices (ECI) were estimated to quantify the degree of socioeconomic inequalities in OHSU. The ECI was calculated for each study year, and subsequently, changes in the ECI between the 2 years were evaluated, both at the general population level and according to each category of the explanatory variables included. These procedures were performed using the "conindex" command, following the methodology proposed by O'Donnell et al.^25^ The value of the ECI can range from −1 to 1, with a positive or negative value indicating whether OHSU is concentrated in rich or poor individuals. Finally, a decomposition analysis of inequality was performed using a multivariable generalised linear model, following the methodology of Wagstaff et al.^26^^,^^27^ Specifically, the decomposition obtains the relative contribution of each variable in the model to the inequality studied.

### Ethical considerations

This study did not require the approval of an ethics committee as it involved the analysis of publicly available, aggregated secondary data that did not allow the identification of the participants evaluated. ENDES was part of the Demographic and Health Surveys (DHS) Program and had central approval by ICF International and in each country of execution. Informed verbal consent was obtained from all respondents, and the data were disseminated in an anonymised form on the microdata website of INEI: https://proyectos.inei.gob.pe/microdatos/. More information on data use and ethical guidelines of the DHS Program is available at https://dhsprogram.com/methodology/Protecting-the-Privacy-of-DHS-Survey-Respondents.cfm.

## Results

A total of 5182 and 4125 older adult participants of the ENDES in 2019 and 2021, respectively, were included in the study (see Figure). The majority of participants in both years belonged to the age group of 60 to 69 years (52.8%, 53.5%), were female (52.5%, 52.7%), and had a primary education level (40.4%, 39.7%). Additionally, most participants lived in urban areas (77.5%, 78.2%), in the Lima Metropolitan region (37.6%, 35.9%), and belonged to the highest wealth quintile (23.9%, 26.9%). The majority of participants had health insurance (83.1%, 86.8%), had no functional limitation (93.2%, 90.9%), and spoke Spanish, Portuguese, or other foreign languages (73.3%, 73.1%) (see Table 1).

**Fig:**
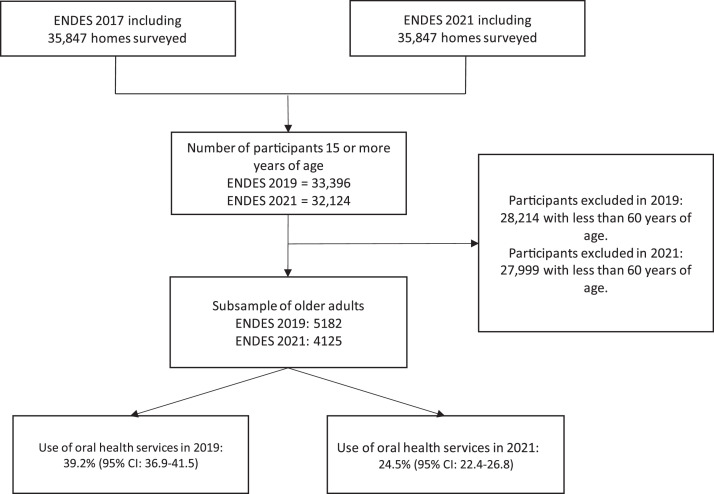
Flowchart of participants included in the study.

**Table 1:** Characteristics of the older Peruvian adults included in the ENDES 2019 and 2021.

Characteristic	2019 (n = 5182)		2021 (n = 4125)	
	No.	% (95% CI)	No.	% (95% CI)
Age group, y				
60–69	2700	52.8 (50.6–54.9)	2323	53.3 (50.9–55.8)
70–79	1647	30.2 (28.3–32.2)	1236	30.0 (27.8–32.3)
≥80	835	17.0 (15.2–19.0)	566	16.6 (14.6–18.9)
Sex				
Female	2808	52.5 (50.2–54.7)	2239	52.7 (50.1–55.3)
Male	2374	47.5 (45.3–49.8)	1886	47.3 (44.7–49.9)
Level of education				
Up to initial education	1105	13.8 (12.6–15.1)	798	14.2 (12.7–15.8)
Primary	2477	40.4 (38.5–42.4)	1873	39.7 (37.3–42.2)
Secondary	922	24.2 (22.3–26.3)	894	26.4 (24.2–28.7)
Higher	678	21.6 (19.7–23.6)	560	19.8 (17.8–21.9)
Area of residence				
Urban	2757	77.5 (76.4–78.6)	2295	78.2 (77.0–79.4)
Rural	2425	22.5 (21.4–23.6)	1830	21.8 (20.6–22.9)
Region of residence				
Metropolitan Lima	541	37.6 (35.9–39.2)	474	35.9 (34.0–37.8)
Rest of the coast	1310	25.6 (24.2–27.1)	1077	25.9 (25.4–28.6)
Highlands	2433	27.2 (25.7–28.7)	1746	26.1 (24.6–27.7)
Jungle	898	9.7 (8.9–10.5)	828	11.1 (9.9–12.2)
Wealth quintile				
Richest	616	23.9 (22.1–25.9)	516	26.9 (24.6–29.2)
Richer	616	20.7 (18.8–22.8)	532	18.7 (16.8–20.7)
Middle	718	17.3 (15.7–18.9)	583	17.5 (15.8–19.4)
Poorer	924	15.9 (14.4–17.4)	750	15.2 (13.9–16.7)
Poorest	2308	22.2 (21.0–23.5)	1744	21.7 (20.4–23.0)
Health insurance				
No	764	16.9 (15.2–18.7)	510	13.2 (11.5–15.1)
Yes	4418	83.1 (81.3–84.8)	3615	86.8 (84.9–88.5)
Limitation				
No	4733	93.2 (91.9–94.2)	3770	90.9 (89.1–92.5)
Yes	449	6.9 (5.8–8.0)	355	9.1 (7.5–10.9)
Language learned in childhood				
Spanish, Portuguese, or other foreign languages	3011	73.3 (71.6–75.0)	2555	73.1 (70.9–75.1)
Quechua or aboriginal languages	2171	26.7 (24.9–28.4)	1570	26.9 (24.9–29.1)

All estimates took into account the weighting and ENDES sample design.

The probability of OHSU in the study population decreased by 37% in 2021 compared to 2019. Significant reductions were observed in all the categories of evaluated variables, except for the population of the jungle region and individuals without health insurance. The greatest decreases in the probability of OHSU were observed in adults aged 80 years or older (51%), those belonging to the lowest wealth quintile (47%), individuals with some functional limitation (53%), and persons whose native language is Quechua or other indigenous languages (47%) (see Table 2).

**Table 2:** Frequencies and adjusted prevalence ratios comparisons of use of oral health services during the last year in older Peruvian adults 2019–2021.*

Characteristic	2019 (n = 5182)		2021 (n = 4125)		aPR (95% CI)	*P* value
	No	Yes	No	Yes		
	No. (%)	No. (%)	No. (%)	No. (%)		
Overall	3439 (60.8%)	1743 (39.2%)	3185 (75.5%)	940 (24.5%)	0.63 (0.56–0.70)	<.001
Age group, y						
60–69	1693 (57.2%)	1007 (42.8%)	1733 (73.4%)	590 (26.6%)	0.62 (0.55–0.70)	<.001
70–79	1108 (63.5%)	539 (36.5%)	967 (74.4%)	269 (25.6%)	0.70 (0.57–0.86)	.001
≥80	638 (67.3%)	197 (32.7%)	485 (83.9%)	81 (16.1%)	0.49 (0.33–0.73)	<.001
Sex						
Female	1866 (59.3%)	942 (40.7%)	1778 (76.7%)	461 (23.3%)	0.57 (0.50–0.66)	<.001
Male	1573 (62.5%)	801 (37.5%)	1407 (74.1%)	479 (25.9%)	0.69 (0.60–0.80)	<.001
Level of education						
Up to initial education	861 (74.5%)	244 (25.5%)	697 (86.3%)	101 (13.7%)	0.58 (0.41–0.81)	.002
Primary	1747 (68.9%)	730 (31.1%)	1506 (80.5%)	367 (19.5%)	0.62 (0.53–0.74)	<.001
Secondary	544 (57.8%)	378 (42.2%)	647 (72.6%)	247 (27.5%)	0.67 (0.55–0.81)	<.001
Higher	287 (40.3%)	391 (59.7%)	335 (61.5%)	225 (38.5%)	0.68 (0.57–0.81)	<.001
Area of residence						
Urban	1643 (56.3%)	1114 (43.7%)	1666 (72.9%)	629 (27.1%)	0.62 (0.55–0.70)	<.001
Rural	1796 (76.5%)	629 (23.5%)	1519 (84.5%)	311 (15.5%)	0.66 (0.56–0.78)	<.001
Region of residence						
Metropolitan Lima	272 (49.0%)	269 (50.9%)	323 (70.4%)	151 (29.6%)	0.57 (0.47–0.70)	<.001
Rest of the coast	813 (64.5%)	497 (35.5%)	822 (78.3%)	255 (21.7%)	0.60 (0.50–0.71)	<.001
Highlands	1691 (68.7%)	742 (31.3%)	1390 (78.1%)	356 (21.9%)	0.70 (0.60–0.81)	<.001
Jungle	663 (74.8%)	235 (25.2%)	650 (78.9%)	178 (21.0%)	0.83 (0.67–1.03)	.095
Wealth quintile						
Richest	262 (40.1%)	354 (59.9%)	317 (63.3%)	199 (36.7%)	0.60 (0.50–0.72)	<.001
Richer	341 (58.2%)	275 (41.8%)	368 (70.5%)	164 (29.5%)	0.70 (0.57–0.86)	.001
Middle	459 (62.0%)	259 (37.9%)	430 (79.5%)	153 (20.5%)	0.55 (0.44–0.70)	<.001
Poorer	622 (69.4%)	302 (30.6%)	592 (82.9%)	158 (17.1%)	0.53 (0.42–0.67)	<.001
Poorest	1755 (78.6%)	553 (21.4%)	1478 (86.3%)	266 (13.7%)	0.64 (0.53–0.76)	<.001
Health insurance						
No	554 (72.5%)	210 (27.5%)	416 (74.8%)	94 (25.2%)	0.91 (0.68–1.21)	.510
Yes	2885 (58.5%)	1533 (41.5%)	2769 (75.6%)	846 (24.4%)	0.59 (0.53–0.66)	<.001
Limitation						
No	3108 (60.5%)	1625 (39.5%)	2896 (74.4%)	874 (25.6%)	0.64 (0.58–0.72)	<.001
Yes	331 (65.7%)	118 (34.3%)	289 (85.9%)	66 (14.0%)	0.47 (0.32–0.70)	<.001
Language learned in childhood						
Spanish, Portuguese, or other foreign languages	1930 (59.5%)	1081 (40.5%)	1930 (73.2%)	625 (26.8%)	0.66 (0.59–0.74)	<.001
Quechua or aboriginal languages	1509 (64.6%)	662 (35.4%)	1255 (81.5%)	315 (18.5%)	0.53 (0.44–0.65)	<.001

⁎ The PR estimations on this table must be interpreted under the corrected *P* value of .002. All estimates took into account the weighting and ENDES sample design and were adjusted for age and sex, except for age and sex when evaluated as the independent variables (sex was adjusted only for age, and age for sex). aPR, adjusted prevalence ratios indicates the comparison of the year 2021 with respect to 2019 for each category of the variables of interest.

Significant reductions were identified in the ECI of OHSU between 2019 and 2021 (difference: −0.1074; *P* = .003). Likewise, significant decreases in the ECI were detected for the age groups of 60 to 69 years and 80 years or older, males, rural populations, persons with a lower wealth quintile, individuals with or without functional limitations, and those with languages learned in childhood such as Spanish, Portuguese, or other foreign languages and Quechua or other indigenous languages (see Table 3).

**Table 3:** Changes in inequalities measured by means of the concentration index of use of oral health services during the last year in older Peruvian adults between 2019 and 2021.

Characteristic	2019		2021		Diff.	*P* value*
	No.	ECI	No.	ECI	2021–2019	
Overall	5182	0.3167	4125	0.2093	−0.1074	.003
Age group, y						
60–69	2700	0.3218	2323	0.1803	−0.1415	.002
70–79	1647	0.2249	1236	0.2785	0.0535	.444
≥80	835	0.4379	566	0.1783	−0.2596	.002
Sex						
Female	2808	0.3193	2239	0.2350	−0.0842	.076
Male	2374	0.3103	1886	0.1828	−0.1276	.018
Level of education						
Up to initial education	1105	0.2709	798	0.1344	−0.1365	.100
Primary	2477	0.1679	1873	0.1103	−0.0577	.285
Secondary	922	0.1787	894	0.1817	0.0029	.969
Higher	678	0.2287	560	0.0612	−0.1676	.061
Area of residence						
Urban	2757	0.2678	2295	0.1992	−0.0686	.122
Rural	2425	0.1719	1830	0.0949	−0.0771	.030
Region of residence						
Metropolitan Lima	541	0.2143	474	0.2009	−0.0135	.867
Rest of the coast	1310	0.2657	1077	0.1564	−0.1093	.047
Highlands	2433	0.2599	1746	0.2175	−0.0425	.405
Jungle	898	0.2201	828	0.2341	0.0140	.808
Wealth quintile						
Richest	616	0.0556	516	0.0363	−0.0192	.837
Richer	616	0.0722	532	0.0598	0.0067	.939
Middle	718	0.1012	583	0.0156	−0.0855	.271
Poorer	924	−0.0382	750	0.0276	0.0658	.274
Poorest	2308	0.1161	1744	0.0397	−0.0764	.020
Health insurance						
No	764	0.2376	510	0.2851	0.0474	.621
Yes	4418	0.3283	3615	0.1999	−0.0128	.001
Limitation						
No	4733	0.3093	3770	0.2271	−0.0822	.032
Yes	449	0.3929	355	0.0265	−0.3665	<.001
Language learned in childhood						
Spanish, Portuguese or other foreign languages	3011	0.3386	2555	0.2304	−0.1082	.015
Quechua or aboriginal languages	2171	0.252	1570	0.0904	−0.1616	.010

All estimates took into account the weighting and ENDES sample design..

⁎ *P* value indicates the probability of nonsignificant change in inequalities according to the concentration index from 2017 to 2021. ECI, Erreygers concentration index; Diff, differences between ECI 2017 and ECI 2021.

The decomposition analysis revealed that the poorest wealth quintile was the primary driver of inequality in both years under evaluation. Specifically, in 2019 the poorest quintile contributed to 51.9% of the inequality, whilst in 2021 this contribution increased to 73.1% (see Table 4).

**Table 4:** Decomposition analysis of inequalities in the use of oral health services during the last year in older Peruvian adults in 2019 and 2021.

Characteristic	2019				2021			
	n = 5182				n = 4125			
	Elasticity	ECI	Absolute contribution	Percentage contribution (%)	Elasticity	ECI	Absolute contribution	Percentage contribution (%)
Age group, y								
60–69	Ref.				Ref.			
70–79	−0.0149	−0.0174	0.0009	0.33	0.0022	−0.0197	−0.0002	−0.08
≥80	−0.0116	−0.0281	0.0019	0.41	−0.0137	−0.0014	0.0001	0.04
Sex								
Female	Ref.				Ref.			
Male	−0.0185	−0.0338	0.0013	0.79	0.0086	−0.0285	−0.0010	−0.47
Level of education								
Up to initial education	Ref.				Ref.			
Primary	0.0146	−0.3550	−0.0128	−6.53	0.0092	−0.3168	−0.0116	−3.57
Secondary	0.0172	0.1976	0.0140	4.43	0.0109	0.1596	0.0069	3.31
Higher	0.0369	0.4310	0.0736	20.07	0.0169	0.4014	0.0272	12.99
Area of residence								
Urban	Ref.				Ref.			
Rural	−0.0002	−0.6416	0.0007	0.19	0.0075	−0.6371	−0.0191	−9.11
Region of residence								
Metropolitan Lima	Ref.				Ref.			
Rest of the coast	−0.0139	0.0300	−0.0016	−0.53	−0.0059	0.0361	−0.0008	−0.41
Highlands	−0.0095	−0.4539	0.0159	5.45	0.0181	−0.4364	−0.0315	−15.07
Jungle	−0.0090	−0.1601	0.0149	1.83	0.0057	−0.1873	−0.0043	−2.06
Wealth quintile								
Richest	Ref.				Ref.			
Richer	−0.0234	0.2595	−0.0294	−7.67	−0.0085	0.2059	−0.0070	−3.33
Middle	−0.0207	−0.0456	0.0055	1.19	−0.0225	−0.0603	0.0054	2.60
Poorer	−0.0262	−0.2518	0.0417	8.34	−0.0270	−0.2522	0.0272	13.00
Poorest	−0.0590	−0.6913	0.1835	51.95	−0.0562	−0.6796	0.1529	73.05
Health insurance								
No	Ref.				Ref.			
Yes	0.1086	0.0204	0.0027	2.80	−0.0149	0.0177	−0.0011	−0.50
Limitation								
No	Ref.				Ref.			
Yes	−0.0001	−0.0588	0.0001	0.01	−0.0092	0.0005	−0.0001	−0.01
Language learned in childhood								
Spanish, Portuguese, or other foreign languages	Ref.				Ref.			
Quechua or aboriginal languages	0.0156	−0.3055	−0.0179	−6.02	−0.0089	−0.2686	0.0096	4.58
Residual			0.0746				0.0565	

All estimates took into account the weighting and ENDES sample design.

ECI, Erreygers concentration index.

## Discussion

This study aimed to examine inequalities in OHSU services amongst elderly Peruvians by comparing data from the ENDES surveys of 2019 and 2021. The study found that the probability of OHSU in the elderly population decreased by 37% in 2021 compared to 2019. The largest declines were observed in adults aged 80 or older, those belonging to the lowest wealth quintile, individuals with functional limitations, and persons whose native language is Quechua or other indigenous languages. Despite the negative socioeconomic impact of the COVID-19 pandemic, a significant reduction in inequalities in OHSU was observed at the national level and in subpopulations such as residents of rural areas, individuals belonging to the lowest wealth quintile, and persons with functional limitations. The poorest wealth quintile was identified as the main contributors to inequality, accounting for 73% of the inequality in OHSU in 2021. The COVID-19 pandemic has widely reduced the probability of OHSU in Peru, disproportionately affecting vulnerable groups. Urgent and well-designed measures are needed to address the unmet demand and improve access to oral health services for the most vulnerable populations.

The probability of OHSU amongst elderly Peruvians significantly decreased in 2021 compared to 2019, which is consistent with findings from a study in the UK that reported a decline in OHSU in the general population of elderly adults during the COVID-19 pandemic.^11^ This decline could be attributed to various factors, such as fear of COVID-19 exposure, reduced availability of oral health services, financial constraints, and limited access to services in rural and remote areas.^28^, ^29^, ^30^ Although the frequency of OHSU in 2021 increased from 16.8% in 2020 to 24.5%, it still falls short of prepandemic levels (39.2%). This may be attributed to the high rate of COVID-19 infections and deaths in Peru during the first half of 2021,^31^ leading to postponed dental visits or only seeking care for emergencies. Moreover, studies have shown that changes in behaviour and psychosocial factors due to the pandemic have also led to a significant decrease in the frequency of brushing, self-perceived need for dental care, and OHSU amongst other population groups, which could also apply to elderly individuals.^32^ Additionally, dental emergencies are perceived as relevant factors for oral health care in the elderly, regardless of their insurance status.^33^ Whilst 7 out of 10 Peruvians have received at least 2 doses of the COVID-19 vaccine, overcoming the worst phases of the pandemic in terms of morbidity and mortality,^34^ strategies are needed to address the negative impact of the pandemic on OHSU amongst the elderly population.

The most vulnerable groups, including older adults in the lowest wealth quintile, those older than 80 years, people with functional limitations, and those whose native language is Quechua or other indigenous languages, experienced the greatest declines in OHSU probability. These findings highlight the persistence of oral health inequalities in Peru, stemming not only from economic factors^35^ but also from structural and health system issues.^36^^,^^37^ For example, dependent individuals may not have sufficient support mechanisms within the health system to access oral health services, further worsening their already poorer oral health status.^38^ On the other hand, language differences between providers and users represent an important barrier to the effective use of health services, especially in rural and remote areas of the country.^39^^,^^40^ This aspect is relevant in Peru since, according to our results, almost 27% of older Peruvian adults learned Quechua and other indigenous languages in childhood. Despite national poverty-reduction policies,^41^ further efforts are necessary to address these inherent health system barriers and achieve more equitable access to oral health services for the elderly population in Peru.

Compared to the results of the 2019 ENDES survey, significant reductions in inequalities in OHSU were observed in 2021. This suggests that the concentration distribution in OHSU in older adults may have been redistributed, resulting in a reduction in the quintiles with higher wealth levels and a closer approximation to equality. Previous evidence has reported a reduction in inequality in OHSU following the establishment of universal health insurance, which is expected given that a larger proportion of the Peruvian population could access dental care through health insurance.^35^ However, the reduction in inequality identified after the pandemic may have resulted from the restrictions on oral health care imposed by international organisations and the Peruvian Ministry of Health.^42^^,^^43^ This scenario may have restricted OHSU to the care of oral conditions that generate a greater perception of need for care by users, reducing preventive and cosmetic care, which is typically performed by a fraction of the population with greater resources. This may have created a false balance in OHSU, which is not perceptible through the analysis carried out. The reductions in inequalities observed at the subgroup level of the population may respond to the same mechanisms, and further research is necessary to understand this "paradox in oral health services utilisation inequalities." Therefore, it is essential to monitor OHSU in the older adult population after the pandemic to determine whether the reduction in inequality persists or returns to its baseline state. Ideally, investigations into the reasons for oral care should also be conducted.

The study found that the lowest wealth quintile was the primary contributor to OHSU inequality in both years evaluated. In 2021, the poorest quintile accounted for 73.1% of the inequality in OHSU amongst older adults in Peru. Previous research has emphasised the importance of socioeconomic status in determining access to oral health services for older adults,^44^, ^45^, ^46^, ^47^, ^48^ and financial barriers likely play a significant role in limiting access for this group, especially for high-cost treatments.^49^ This problem has been exacerbated by the economic impact of the pandemic, which appears to have disproportionately affected this group, who in 2019 accounted for a lower percentage (51.9%) of the inequality in OHSU. In this regard, Peruvian families in the lowest wealth quintile tend to allocate almost 80% of per capita family income to food and rent, with less than 10% allocated to health care.^50^ To address these disparities, effective health policies and programs are urgently needed to increase access to oral health services for vulnerable populations in Peru.

The present study has several limitations that should be considered when interpreting its results. Self-reported data may be prone to recall bias and data collection errors by surveyors. Additionally, social desirability bias could have affected the measurement of OHSU. Moreover, as the study is cross-sectional, causal relationships cannot be established. Furthermore, the measurement of the use of oral health services did not differentiate between different types of treatment, which could have influenced the measurement of this variable during the pandemic when treatments were limited to dental emergencies and urgencies. Despite these limitations, the study also has several strengths. The analysis of inequality considered the well index, which is a more sensitive indicator of socioeconomic status than income levels, particularly in older adults.^51^^,^^52^ Furthermore, the data were obtained from a nationally representative survey with technical assistance from the DHS Program, which has extensive experience in implementing population surveys in more than 90 countries worldwide.^53^ Finally, the study provides current information on inequalities in the use of oral health services in older adults in Peru, which can be useful for research and decision-making purposes.

## Conclusions

This study highlights the challenges facing dental public health in Peru regarding OHSU inequalities amongst older adults. Improving accessibility to oral health care services is crucial, especially for those in lower wealth quintiles, older than 80 years old, with functional limitations, or who speak Quechua or other indigenous languages. Multisectoral policies are needed to address not only the economic determinants of health but also the structural, cultural, and geographic issues of the health care system. Additionally, promoting oral health and educating the elderly on prevention and dental care can increase awareness and reverse pandemic-related declines in utilisation. Although we identified a reduction in inequalities in OHSU, this could solely be due to usage patterns resulting directly from the health care restrictions imposed by national and international health organisations, despite the economic impact of the pandemic. We have named this phenomenon "the paradox of inequalities in oral health service utilisation," but further research is needed to better understand this paradox.

## Conflict of interest

None disclosed.
